# Alpha neurofeedback training improves visual working memory in healthy individuals

**DOI:** 10.1038/s41539-024-00242-w

**Published:** 2024-04-18

**Authors:** Wenbin Zhou, Wenya Nan, Kaiwen Xiong, Yixuan Ku

**Affiliations:** 1 School of Psychology, Shanghai Normal University, Shanghai, China; 2 Zhengzhou Shuqing Medical College, Zhengzhou, China; 3 The Research Base of Online Education for Shanghai Middle and Primary Schools, Shanghai, China; 4 Guangdong Provincial Key Laboratory of Brain Function and Disease, Center for Brain and Mental Wellbeing, Department of Psychology, Sun Yat-sen University, Guangzhou, Guangdong, China; 5 Peng Cheng Laboratory, Shenzhen, Guangdong, China

**Keywords:** Working memory, Brain-machine interface, Object vision

## Abstract

Neurofeedback (NF) training is a closed-loop brain training in which participants learn to regulate their neural activation. NF training of alpha (8–12 Hz) activity has been reported to enhance working memory capacity, but whether it affects the precision in working memory has not yet been explored. Moreover, whether NF training distinctively influences performance in different types of working memory tasks remains unclear. Therefore, the present study conducted a randomized, single-blind, sham-controlled experiment to investigate how alpha NF training affected the capacity and precision of working memory, as well as the related neural change. Forty participants were randomly and equally assigned to the NF group and the sham control group. Both groups received NF training (about 30 min daily) for five consecutive days. The NF group received alpha (8–12 Hz) training, while the sham control group received sham NF training. We found a significant alpha increase within sessions but no significant difference across sessions. However, the behavioral performance and neural activity in the modified Sternberg task did not show significant change after alpha NF training. On the contrary, the alpha NF training group significantly increased visual working memory capacity measured by the Corsi-block tapping task and improved visual working memory precision in the interference condition in a color-recall task. These results suggest that alpha NF training influences performance in working memory tasks involved in the visuospatial sketchpad. Notably, we demonstrated that alpha NF training improves the quantity and quality of visual working memory.

## Introduction

Visual working memory (WM) is defined as the active maintenance of visual information to serve the needs of ongoing tasks^1^. It is commonly separated into three distinct phases: encoding, maintenance, and retrieval^2^. Current research suggests that visual WM has limited memory resources and can flexibly allocate them to different stored items. The quality (precision) and the quantity (number) of items are two indexes that describe the visual WM ability. As the number of items that need to be remembered increases, the precision of stored items decreases^3^. Visual WM ability is the basis of many high-level cognitive functions, such as problem-solving, reasoning, decision-making, and goal-oriented behavior^4^. The decline in visual WM may lead to an imbalance in the ability to accept visual information and the ability to pay attention. Also, WM performance declines with age^5^, and the decline of visual WM ability causes reduced intellectual function^1^. Therefore, it is important to improve visual WM ability.

Visual WM is based on the continuous neural activity of a complex cerebral cortex network, which includes the frontal, parietal, occipital, and temporal lobes. In visual WM, alpha, beta, and gamma play essential roles^6,7^. More specifically, alpha activity plays a central role in the active storage of information in visual WM^8^. In addition, the desynchronization of pre-stimulus alpha predicts recall precision^9^ and correlates with subjective confidence in discrimination accuracy in WM tasks^10^. In terms of different phases of WM process, it has been reported that alpha occurs event-related desynchronization (ERD) in the encoding phase and event-related synchronization (ERS) in the maintenance phase^11^. To sum up, the above findings indicate the importance of alpha activity in visual WM.

Consequently, many studies have applied EEG neurofeedback (NF) to up-regulate alpha activity for visual WM enhancement. NF utilizes the operant conditioning principle to regulate brain activity of interest for cognition/behavior enhancement. During NF training, the EEG signals are recorded from the scalp, and relevant EEG feature is converted into auditory or/and visual information that individuals can understand. According to this information, individuals learn to regulate their EEG activity to improve corresponding cognitive function and behavioral performance^12,13^. Some studies have found that NF training on alpha activity can effectively improve the WM ability of healthy individuals. For example, Nan et al.^14^ found significant improvement in WM performance in the alpha NF group compared to the non-NF control group. Notably, the increase in upper alpha by NF was positively correlated with WM enhancement. In addition, Hsueh et al.^15^ reported that alpha NF significantly improved WM compared to the random frequency band NF training (i.e., sham-NF). A recent systematic review and meta-analysis study concluded that alpha NF training positively affects WM^16^.

However, most of these NF studies only measured the quantity (i.e., the number of items) of visual WM using different tasks, such as the N-back task^17–19^, digit span task^14,20^, recall task^21^, and mental rotation test^22^. Another critical visual WM index, i.e., quality (precision), must be considered in NF research. Furthermore, previous alpha NF studies that successfully improved WM ability were unable to clearly distinguish the changes in alpha at specific visual WM phases due to the sequential presentation of the stimuli, which causes encoding and maintenance operations to occur in parallel^18,23–25^. To our knowledge, how alpha NF training impact neural activity in the WM process has been rarely explored.

Therefore, this randomized single-blind sham-controlled study aimed to investigate the influence of alpha (8–12 Hz) NF training on visual WM from quantity, precision, and neural activity in different visual WM phases. The color-recall task and the Corsi-block tapping task measured the precision and quantity of visual WM^26^. Given that the modified Sternberg task can effectively separate the visual WM process into encoding, maintenance, and retrieval phases^27^, the NF training effect on neural activity in the visual WM process was examined by pre-stimulus alpha power and alpha ERS/ERD in encoding and maintenance phases in the modified Sternberg task.

## Results

### EEG results

#### Alpha changes during NF training

**Fig. 1:**
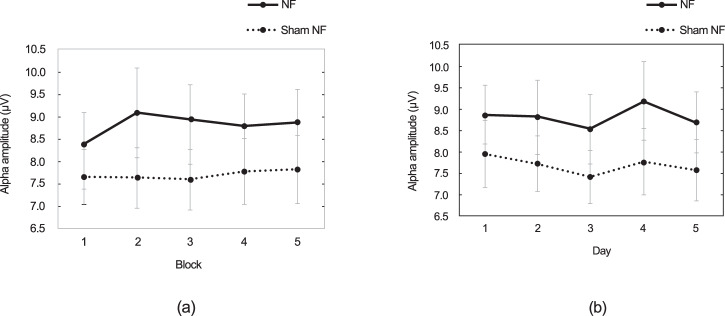
The mean alpha amplitude during training for both groups. **a** mean alpha in each block (**b**) mean alpha in each session. The error bars indicate *SE*.

Figure 1 presents the mean alpha amplitude time course in each block and session for both groups. Regarding the alpha within sessions, the repeated-measures ANOVA revealed that Group had no significant main effect (*F* (1,38) = 1.18, *p* = 0.284), whereas the main effect of the Block was marginally significant (*F* (4,35) = 2.54, *p* = 0.057, η_p_^2^ = 0.225). More importantly, Group $\times$ Block has a significant interaction (*F* (4,35) = 3.29, *p* = 0.022, η_p_^2^ = 0.273), indicating that alpha changes across blocks significantly differed between the NF training group and the sham control group. Further analysis found a significant increase in alpha amplitude in the last three blocks (blocks 3 to 5) compared to Block 1 in the NF group, which was not the case in the sham control group.

For the alpha across sessions, the repeated-measures ANOVA revealed a marginally significant main effect of the day (*F* (4,35) = 2.54, *p* = 0.057, η_p_^2^ = 0.225). In contrast, neither Group $\times$ Day interaction (*F* (4,35) = 0.22, *p* = 0.924) nor the main effect of group (*F* (1,38) = 1.21, *p* = 0.278) showed significance.

#### Alpha ERSP and pre-stimulus alpha in the modified Sternberg task

Table 1 presents the pre-stimulus alpha and the alpha ERSP in the encoding and maintenance phases of the modified Sternberg task in pretest and posttest 1. It can be observed that the alpha ERSP in the encoding and maintenance phases was positive, suggesting that the phenomenon of ERS happened. In addition, the sham control group showed a slight ERS reduction from pretest to posttest 1, whereas the NF training group had more ERS reduction in posttest 1.

**Table. 1:** The difference of alpha activity after NF training in two groups

Feature	Group	Pretest (*M* ± *SE*)	Posttest 1(*M* ± *SE*)
ERSP in the encoding phase	NF	11.286 ± 5.73	5.928 ± 6.26
	Sham control	10.034 ± 5.73	6.019 ± 6.26
ERSP in the maintenance phase	NF	11.988 ± 6.66	7.932 ± 8.47
	Sham control	15.277 ± 6.657	9.659 ± 8.47
Pre-stimulus alpha	NF	20.624 ± 0.804	21.097 ± 0.781
	Sham control	21.005 ± 0.804	21.15 ± 0.781

The repeated-measures ANOVA showed no significant Group$\times$Time interaction in the alpha ERSP in the encoding stage (*F* (1,38) = 0.03, *p* = 0.861) and in the maintenance stage (*F* (1,38) = 0.05, *p* = 0.833). Similarly, the pre-stimulus alpha activity had no significant Group $\times$ Time interaction (*F* (1,38) = 0.49, *p* = 0.486). These results together indicated that alpha NF training had no impact on the alpha activity in the modified Sternberg task compared to the sham control group.

### Behavioral results

**Fig. 2:**
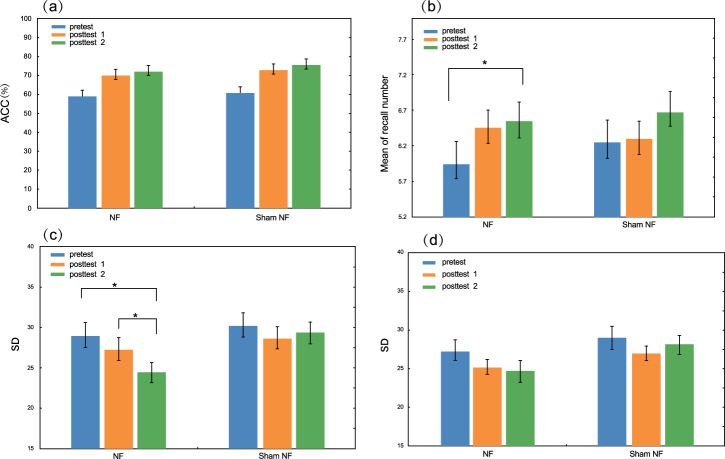
Behavioral results. **a** The mean accuracy of modified Sternberg task in each group. **b** The performance of Corsi-block tapping task. **c** Color-recall task performance of the two groups in the interference condition. **d** Color-recall task performance of the two groups in the non-interference condition. The error bars indicate SE.

Figure 2 presents the performances of the modified Sternberg task, Corsi-block tapping task, and Color-recall task in the pretest, posttest 1, and posttest 2.

For the ACC of the modified Sternberg task, repeated-measures ANOVA revealed a significant main effect of Test (*F* (2,37) = 39.38, *p* < 0.001, η_p_^2^ = 0.509), which was not found for the Group (*F* (1,38) = 0.60, *p* = 0.442). Also, there was no significant Group $\times$ Test interaction (*F* (2,37) = 0.14, *p* = 0.873), indicating that the alpha NF training group did not improve the performance of the modified Sternberg task compared with the sham control group.

Regarding the Corsi-block tapping task score (Fig. 2b), Friedman’s rank test showed marginal significance in the alpha NF group (${\chi }^{2}(2)=5.69,{p}=0.058)$. Further Wilcoxon signed rank test with Bonferroni correction revealed that the NF group had a significant enhancement in posttest 2 (M = 6.55, SE = 0.27) compared to pretest (M = 5.95, SE = 0.32; *z* = 2.49; *p* = 0.039), but no significant change between pretest and posttest 1 (M = 6.45, SE = 0.25; *z* = 1.90; *p* = 0.174) or between posttest 2 and posttest 1(*z* = −0.49; *p* = 1). Regarding the sham control group, Friedman’s rank test did not reveal significance (${\chi }^{2}(2)=2.77,{p}=0.25)$. This result indicated that alpha NF training could improve the WM capacity, reflected by the lasting effect measured on 7 days after the end of NF training.

Figure 2c presents the mean absolute error in the interference condition for both groups. It can be observed that the alpha NF group decreased the mean absolute error from the pretest (M = 28.91, SE = 1.64) to posttest 1 (M = 27.24, SE = 1.45) and further reduced in posttest 2 (M = 24.42, SE = 1.22). Friedman’s rank test showed significance in the interference condition in the alpha NF group $({\chi }^{2}\left(2\right)=16,{p} \,<\, 0.001)$. Further Wilcoxon signed rank test with Bonferroni correction revealed a significant difference between any two tests except for posttest 1 and pretest (posttest 1 vs. pretest: *z* = −1.91, *p* = 0.336; posttest 2 vs. pretest: *z* = −2.84, *p* = 0.03; posttest 2 vs. posttest 1: *z* = −2.78, *p* = 0.03). For the non-interference condition in the alpha NF group, Friedman’s rank test did not show significance (${\chi }^{2}\left(2\right)=3.11,{p}=0.211)$. For the sham NF group, Friedman’s rank test revealed no significance for either interference condition (${\chi }^{2}\left(2\right)=0.90,{p}=0.638)$ or non-interference condition (${\chi }^{2}\left(2\right)=3.10,{p}=0.212)$. The above results indicated the improved visual WM precision by alpha NF training.

## Discussion

By a single-blind sham-controlled design, this study investigated alpha up-regulation NF training effects on WM regarding both neural activity and behavioral performance. The alpha NF group revealed a significant increase in alpha amplitude within sessions, which was not found in the sham control group. In addition, the group receiving genuine alpha NF training showed significant improvement in the Corsi-block tapping and Color-recall tasks compared with the sham control group.

We found that the alpha NF group could significantly increase alpha amplitude within sessions compared to the sham control group, indicating successful short-term NF learning. However, such an increase could not be maintained across sessions, i.e., unsuccessful long-term NF learning. One of the possible reasons may be the feedback presentation. Most studies reported that alpha power could be up-regulated via visual feedback^15,18,24^. For example, Pei et al.^25^ reported a significant alpha increase after five sessions of alpha up-regulation NF training, where a “face” picture was taken as visual feedback material. The “face” can be changed into “smiling” or “crying” when the target frequency power is greater or lower than the threshold. In our study, participants were asked to learn to increase their alpha activity through a blue bar in NF training. However, after the experiment, some participants reported feeling bored when doing NF training. Motivational factor is one predictor of NF success, and the learning efficiency of NF training will be improved by the participant’s strong motivation^28^. The uninteresting feedback interface may cause low motivation and reduce the learning efficiency in NF training. In addition, the small session number may also be the reason for the failure of long-term NF learning, since successful long-term NF learning could be obtained by more than five training sessions^14,18,23,24,29,30^.

Nevertheless, we found that alpha increase within sessions was associated with significant improvement in WM capacity from the pretest to the posttest 2 performed seven days after the end of NF training. In line with Kober et al.^31^, increased alpha within sessions but not across sessions by NF training improved WM capacity assessed by Corsi-block tapping task in both post-stroke victims and healthy controls.

Besides WM capacity, we were also interested in whether alpha NF training could enhance visual precision, which has been rarely answered yet in the literature. We found that the alpha NF training group significantly improved visual precision under the interference condition from pretest to seven days after NF training, which was not observed in the non-interference condition. During the WM process, the increased alpha activity can inhibit the interference of irrelevant information^11,32,33^. Bonnefond and Jensen^34^ found that more alpha power appeared under the strong interference condition than the weak one in the modified Sternberg task. In other words, less interfering information requires less alpha to suppress irrelevant information. In our work, participants in the non-interference condition had less information to remember and were not distracted by irrelevant information. In this case, the participant does not need more alpha to suppress the interference of irrelevant information. Thus, the precision performance under the non-interference condition had no significant change following alpha NF training.

On the other hand, under the interference condition, irrelevant information appears on the opposite side of the cue. In this case, vigorous alpha activity acts as an inhibitory function to suppress irrelevant information, which explains the increased precision after alpha enhancement NF training. The result also supports the idea that alpha can inhibit the interference of irrelevant information in visual WM processing. Future research is desirable to investigate how alpha NF training influences neural activity during the color-recall task to better understand the neural effects of NF training.

Regarding the modified Sternberg task, neither the behavioral performance nor the neural activities (including alpha ERSP and pre-stimulus alpha) showed significant change following alpha NF training, i.e., alpha NF training had no impact on the modified Sternberg task performance. This result is consistent with Hsueh et al.^15^, where alpha NF training did not enhance WM performance measured by the backward digit span task and operation span task compared to the sham control group. However, Zoefel et al.^22^ showed WM improvement measured by mental rotation through alpha NF training. The above contradictory results may be due to the WM tasks requiring different WM storage systems (the visuospatial sketchpad and the phonological loop), where the role of alpha activity has been shown to differ. More specifically, alpha activity tends to occur during tasks that require maintenance of simultaneously presented visual or spatial information that involves visuospatial sketchpad^35^. Since the mental rotation task presented visual-spatial information, it was reasonable to improve mental rotation performance by alpha NF training^22^. Unlike the visual-spatial task, the backward digit span task and operation span task in Hsueh et al.^15^, and the letter information in the Sternberg task in our work involved the phonological loop. In our experiments, the modified Sternberg task, which requires participants to memorize letters, activated the phonological loop to support the maintenance of WM items through subvocal rehearsal^35^. This idea also explains why alpha NF training showed different training outcomes on WM tasks, i.e., the Sternberg task involved phonological loop whereas the color recall task and Corsi-block tapping task involved visuospatial sketchpad.

Amongst the limitations of this study, we only achieved short-term alpha enhancement without across-session enhancement in the NF group compared to the sham control group. In addition, we adopted a fixed alpha band (8–12 Hz) as the NF training target, which did not account for inter-individual differences in alpha frequency. Future NF studies can apply the personalized alpha frequency band for NF protocol optimization. Despite the above limitations, our findings suggest alpha NF training benefits for visual WM quantity and precision.

## Methods

### Participants

The sample size was calculated based on the G*Power program by setting α-error = 0.05, power = 0.8, and effect size *f* = 0.25. The results showed that 36 participants would be required. Given the likelihood of withdrawal during the study, 40 healthy volunteers (mean ± SD age: 23.83 ± 1.26) from the Shanghai Normal University were included. Participants were randomly and equally assigned to either the alpha NF group (7 males and 13 females, mean ± SD age: 24.1 ± 1.21) or the sham control group (9 males and 11 females, mean ± SD age: 23.55 ± 1.28). The experimental protocol was carried out by the Declaration of Helsinki and approved by the Shanghai Normal University ethics committee. Informed consent was provided and signed by all participants before the experiment. All participants reported normal or corrected-to-normal vision, no history of psychiatric disorders or use of psychotropic medications, and had not participated in an NF study in the past. After the experiment, all of them got paid for their participation.

### Experimental design and procedure

**Fig. 3:**
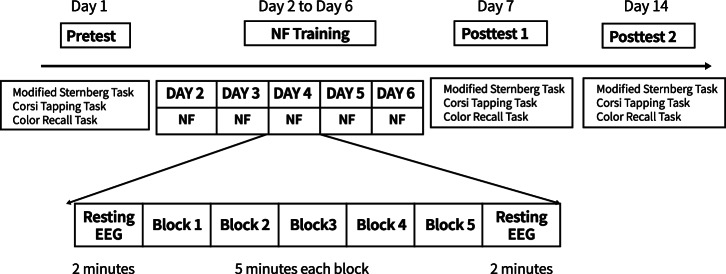
Experimental procedure. There were 5 NF training days, and each training day consisted of five 5-min training blocks and two 2-min resting EEG recordings. The WM performance was assessed before NF training, at the end of NF training, and at 7 days of follow-up.

The study adopted a single-blind, randomized, sham-controlled design. The experiment consisted of four stages within 2 weeks (Fig. 3): (1) pretest on Day 1; (2) five NF training sessions on days 2 to 6; (3) posttest 1 on Day 7; (4) posttest 2 on Day 14.

In the pretest and posttest 1, the participants performed three visual WM tasks, including a modified Sternberg WM task^36^, a Corsi-block tapping task, and a color-recall task. The EEG signals were recorded during the modified Sternberg WM task. After the pretest, five NF training sessions were conducted, with one session per day. Posttest 2 was implemented to explore whether the effect of NF training on behavioral task performance could be maintained, in which only three visual WM tasks were performed without EEG collection.

During each test and NF training session, participants were seated comfortably in a dimly lit and quiet experimental room. Computer-based tasks and NF training were presented on a Dell Computer with a Dell 24-inch monitor placed at a viewing distance of approximately 60 cm.

### EEG data acquisition

On Day 1 and Day 7, the EEG data during the modified Sternberg task were recorded by a 64-channel Neuroscan system, and electrode locations were according to the international 10–20 system. The ground was located at the area between FPz and Fz. The reference electrode was placed on the left mastoid. The impedance for all electrodes was kept below 5 kΩ. The signals were amplified through an amplifier (Neuroscan Amps2) with a sampling rate of 500 Hz. A 0.05–100 Hz bandpass filter was enabled in the amplifier to filter out low-frequency and high-frequency noise.

### NF training protocol

Five NF sessions (five 5-min blocks in each session) were conducted using the Nexus system and BioTrae+ software (NeXus-10 Mark II BioTrace + software (2018A); Mind Media BV, Herten, the Netherlands). During NF training, the EEG signals were recorded from the Pz position with a sampling rate of 256 Hz, the reference electrode was located at the right mastoid, and the ground electrode was placed at the left mastoid. Four electrodes were used to record bipolar, bidirectional electrooculogram (EOG). Electrode impedances were kept below 5 kΩ.

Each participant completed one NF session with five blocks per day from 2nd to the 6th day. Before and after each session, a 2-min fixation cross was present to conduct start and end baselines of resting alpha activity. For the online calculation of the feedback feature during NF training, an infinite impulse response (IIR) Butterworth bandpass filter (8–12 Hz, 3rd order) was applied. The root mean square (RMS) of the resulting signal with an epoch size of 0.25 s was computed to obtain the alpha amplitude as the feedback feature. Based on our NF training experiences, the threshold for the first block in each session was set at 95% of the baseline alpha value. If the percentage of time alpha above the threshold exceeded 60% in the previous block, the threshold would be increased by 0.5 in the next block. In contrast, if the percentage was below 40%, the threshold would be decreased by 0.5 in the next block.

**Fig. 4:**
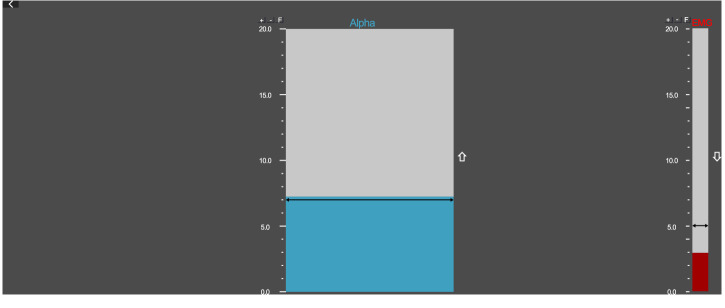
The feedback interface. The blue bar represents the real-time alpha amplitude at the training location Pz. The red bar represents the real-time high frequency artifacts. Data were discarded when the high frequency artifact was above 5 μV.

Feedback was presented visually in a blue bar (Fig. 4), whose height was controlled by the real-time alpha amplitude. Participants were asked to keep the blue bar as high as possible. The high-frequency artifacts reflected by the EEG amplitude from 75 to 100 Hz were also monitored in real-time (the red bar on the right side of the feedback interface), and the subjects needed to keep it below 5 μV. The NF training group trained their real-time alpha amplitude. The sham control group completed the same experimental procedure and received the same instructions as the NF training group. However, the feedback signals were pre-recorded by one participant in the NF training group. None of the sham control group participants reported finding themselves receiving the sham NF.

Participants were instructed to find effective mental strategies to keep the blue bar as high as possible, but no specific strategies were provided. After each training block, there was a short break so that the participants had rest, during which their mental strategies were recorded. The results of the strategies are presented in the Supplementary Table 1.

### Behavior task

#### Modified Sternberg task

**Fig. 5:**
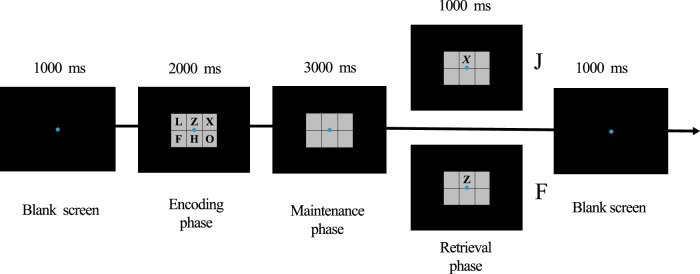
Modified Sternberg task. The task separates the WM process into encoding, maintenance, and retrieval phases.

Modified Sternberg task decomposes the WM process into encoding, maintenance, and retrieval stages. Analyzing the changes in the individual’s EEG in different memory stages makes it possible to understand the NF training effect on WM-related neural activity^36^. This task was presented using E-Prime 2.0 software. The trial procedure is shown in Fig. 5. First, a fixation point appeared on the screen for 1000 ms, and participants were asked to pay attention to the fixation point. Then in the encoding phase, there were six small squares on the screen, each with a different letter (2000 ms). Participants were required to remember the letter in each square. After that, the letters in the squares disappeared for 3000 ms in the maintenance phase. Then in the retrieval phase, the six squares were displayed again on the screen, but only one square contained a letter. Participants needed to determine whether the letter was identical to the previous letter in the same square. If identical, the participants pressed the “F” key with their left hand. Otherwise, they pressed the “J” key with their right hand. The next trial would be presented if the participant did not respond within 1000 ms. Before the formal experiment, participants needed to complete five practice trials to ensure they understood the rules. The formal experiment consisted of two blocks, with 48 trials in each block and a short break between two blocks. Task accuracy (ACC) was regarded as the behavioral indicator of WM performance.

#### Corsi-block tapping task

**Fig. 6:**
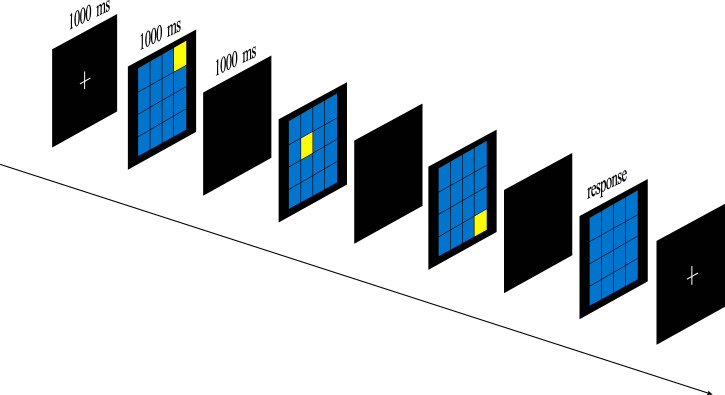
Corsi-block tapping task. This task measures the visual–spatial WM span.

The Corsi-block tapping task was used to measure the visual-spatial WM span. As shown in Fig. 6, a sequence of 3 to 16 identical spatially separated blocks is highlighted, and the participants had to reproduce it in forward order. Each sequence had three trials. If the sequence were reproduced correctly in more than one trial, the following sequence would be increased by one block; if not, the task would be terminated. The participants were allowed to practice twice before formal experiments. The sequence at the end of the experiment represented the task score.

#### Color-recall task

**Fig. 7:**
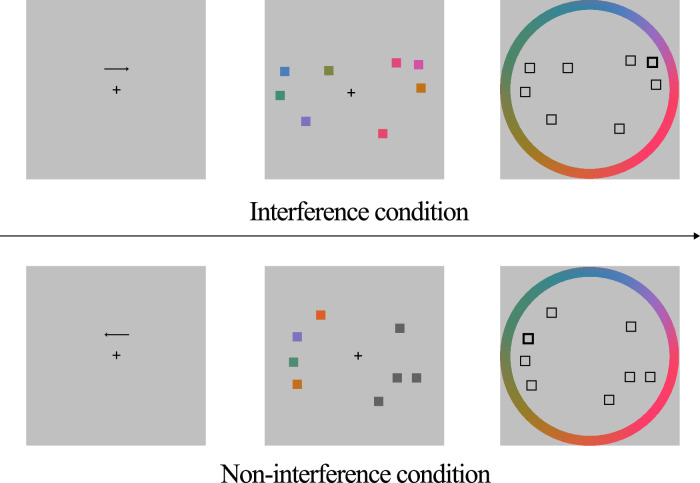
Color-recall task. Top: the interference condition. Down: the non-interference condition.

Color-recall task was presented using MATLAB software (The Mathworks, Natick, MA, USA). This task used the angle difference between the color position of the participant’s response and the correct position of the stimulus color to estimate the participant’s visual WM precision. The indicators of color-recall task can sensitively reflect the differences in the precision of visual WM between individuals^3,37^. This task had three sessions, and each session consisted of 72 trials. The trial procedure is shown in Fig. 7. First, the screen showed “+”, and “←” or “→” appeared randomly above the “+”. The arrow indicates the direction in which the stimulus appears. After 300 ms, four squares with random positions will appear on the left and right of the screen simultaneously. Participants need to pay attention to the stimuli that appear in the direction of the arrow and try to remember the colors of the four squares. The squares on the other side of the screen may be colored (interference condition, a total of 108 trials) or uncolored (non-interference condition, a total of 108 trials). There were also 180 color values with equal space on the screen in the reaction stage, as shown in Fig. 7. Participants were supposed to recall the color remembered before and choose the right color ring with the mouse. Participants practiced for 12 trials before the formal experiment and had a break between sessions. The behavioral index of the color-recall task is mean absolute error calculated by Eq. (1) that represents the visual WM precision. It describes the difference between the color reported by the participant and the correct target color. In the statistical analysis, a Gaussian distribution model was used to exclude the influence of the random response trials on the results^3^. A lower mean absolute error means that the participant has higher visual precision.

1 $${Mean}\,{absolute}\,{error}=\frac{{\rm{|}}{color}\,{reported}-\,{correct}\,{target}\,{color}{\rm{|}}}{{trial}\,{number}}$$

### EEG offline processing

The mean alpha amplitude in each NF block during each training session that had eliminated high-frequency and EOG artifacts was exported from BioTrace+ software.

The EEG data in the modified Sternberg task was processed offline using custom scripts and the EEGLAB toolbox^38^ running under MATLAB software. The unnecessary tracks and electrodes, including M1, M2, HEO, VEO, CB1, and CB2, were first rejected. Then, a bandpass filter (1–20 Hz) was applied. After that, the bad channels were determined and removed through Artifact Subspace Reconstruction (ASR)^39^ and visual inspection. The same method was used to detect and correct the high-amplitude bursts with the 500 ms window standard deviation over 20. Then, the signals were segmented based on the picture onset (−1000 ms to 5000 ms) in the encoding and maintenance stages. Independent component analysis (ICA)^40^ was performed to reject eye, heart, and muscle artifacts. The EEG data was then re-referenced to the average reference, and only epochs with correct responses were selected for analysis.

The Morlet wavelet transforms with a 3-cycle wavelet was applied at the Pz position. Then, the mean pre-stimulus power throughout the baseline period (from −1000 ms to 0 ms) and the event-related spectral perturbation (ERSP) during the encoding and maintenance stage for the alpha band (8–12 Hz) were computed. The ERSP was calculated based on Eq. (2), where active refers to the event-related alpha amplitude (from 0 to 2000 ms in the encoding stage and from 2000 ms to 5000 ms in the maintenance stage). Reference refers to the EEG amplitude at baseline (from −800 ms to −200 ms). When the ERSP is positive, it indicates that event-related synchronization (ERS) occurs. Otherwise, event-related desynchronization (ERD) occurs.

2 $${ERSP} \% =\frac{({\rm{Active}}-{\rm{Reference}})}{{\rm{Reference}}}\times 100$$

### Statistical analysis

The statistical analysis was conducted using SPSS 26.0 and MATLAB R2023b. The statistical tests were all two-tailed, and the significance level was set to 0.05. Initially, we conducted the Shapiro–Wilk normality test for all experimental data to examine the normality of the distributions. Data that met normal distribution were further analyzed using ANOVA, and data that did not meet normal distribution were assessed using a non-parametric test.

We first investigated the alpha amplitude change during the NF training period. For alpha change within sessions, 2 (Group: the NF group, the sham control group) $\times$ 5 (Block: blocks 1 to 5) mixed-design repeated-measures ANOVA was applied to the mean alpha amplitude in each block. Regarding the alpha change across days, 2 (Group: the NF group, the sham control group) $\times$ 5 (Day: days 1 to 5) mixed-design repeated-measures ANOVA was applied to the mean alpha amplitude of each session.

For the alpha ERSP and pre-stimulus alpha in the modified Sternberg task, 2 (Group: the NF group, the sham control group) $\times$ 2 (Test: pretest, posttest 1) mixed-design repeated-measures ANOVA was applied to examine how NF training impacted the event-related neural activity and pre-stimulus alpha.

Regarding the behavioral analyses, 2 (Group: the NF group, the sham control group) $\times$ 3 (Test: pretest, posttest 1, and posttest 2) mixed-design repeated-measures ANOVA was applied for the ACC in the modified Sternberg task.

In the above ANOVA analyses, post hoc pairwise comparisons with Bonferroni correction were applied in case of a significant interaction.

The Shapiro–Wilk normality test showed that the Corsi-block tapping task and Color-recall task data did not meet normal distribution. Consequently, Friedman’s rank test that included all three tests was conducted for each task measure. In case of significance or marginal significance, further pairwise comparison was conducted by Wilcoxon signed rank test with Bonferroni correction for type I error control.

### Reporting summary

Further information on research design is available in the Nature Research Reporting Summary linked to this article.

### Supplementary information

Supplementary Materials

Reporting Summary

## Data Availability

The data supporting the findings of this study are available from the corresponding authors upon request. There are no limits on data sharing.

## Code availability

The code data used in this study are available from the corresponding authors upon request for academic purposes.
