# Supplementary material for: Alpha neurofeedback training improves visual working memory in healthy individuals
Source: NPJ Sci Learn. 2024 Apr 18;9:32. doi: 10.1038/s41539-024-00242-w (PMC11026515; doi:10.1038/s41539-024-00242-w)
Supplement: Supplementary file 1 — Supplementary Materials [file 41539_2024_242_MOESM1_ESM.pdf]

Mental strategies in the NF group

We recorded all mental strategies during training to distinguish which strategies were effective in increasing alpha amplitude. In our experiment, there were 20 subjects in the NF group, and each had five blocks per day for five days, generating a total of 500 mental strategies. We want to know which strategies are effective. To investigate this question, when alpha was greater than the threshold for more than 55% of the total training time of a block, we artificially defined this strategy as an effective strategy. We counted all the effective mental strategies of the NF group according to the frequency of use from high to low, as shown in Supplementary table 1. The effective mental strategies appeared 161 times. Among them, the strategies of relaxing and thinking nothing appeared 83 times, accounting for about 52% of the effective strategies, followed by recollection. Positive memory appeared 15 times, accounting for about 9% of effective strategies.

Supplementary table 1. Proportion of effective mental strategies during alpha NF training.

Effective mental strategies	Number of times	Percentage
Relax	83	52%
Recall positive memory	15	9%
Expect the blue bar to rise	13	8%
Recall negative memory	11	7%
Fantasy future	11	7%
Hot-blooded novel	10	6%
Feel the body	5	3%
Daze	4	2%
Doze	4	2%
Imagine singing	3	2%
Recall memory with no feeling	2	1%
